# Eccentric and Isometric Shoulder Rotation Strength and Range of Motion: Normative Values for Adolescent Competitive Tennis Players

**DOI:** 10.3389/fspor.2022.798255

**Published:** 2022-02-17

**Authors:** Fredrik Johansson, Martin Asker, Andreas Malmberg, Jaime Fernandez-Fernandez, Anna Warnqvist, Ann Cools

**Affiliations:** ^1^Tennis Research and Performance Group, Department of Health Promotion Science, Sophiahemmet University, Stockholm, Sweden; ^2^Scandinavian College of Naprapathic Manual Medicine, Stockholm, Sweden; ^3^Handball Research Group, Department of Health Promotion Science, Sophiahemmet University, Stockholm, Sweden; ^4^Department of Physical Activity and Sports Sciences, Universidad de León, León, Spain; ^5^Division of Biostatistics, Institute of Environmental Medicine, Karolinska Institute, Stockholm, Sweden; ^6^Department of Rehabilitation Sciences, Faculty of Medicine and Health Sciences, Gent, Belgium

**Keywords:** shoulder, hand-held dynamometry, tennis, adolescent, normative database, range of motion, strength

## Abstract

The aim of this cross-sectional study was to investigate isometric internal rotation (IR), external rotation (ER), abduction (ABD), and eccentric external rotation (eccER) shoulder strength and rotational range of motion (ROM) in adolescent male and female competitive tennis players. Additional aims of the study were to provide a tennis-specific normative database based on a large sample of players to deepen the knowledge regarding shoulder strength and ROM for adolescent competitive tennis players, and to discuss differences based on sex, age, and level of play. Shoulder strength and ROM was assessed in 301 adolescent competitive tennis players, 176 boys and 125 girls with a mean age of 14.6 and 14.4 years, respectively. Outcome variables of interest were isometric IR and ER strength, ABD strength, eccER shoulder strength, intermuscular strength ratios ER/IR and eccER/IR, IR ROM, ER ROM, and total range of motion (TROM). A General Linear Model two-way ANOVA was used to analyze differences in sex, age, and level of play. The findings of this study demonstrated age, side, and sex differences in the shoulder isometric strength, the eccER strength and ROM in adolescent competitive tennis players. Furthermore, when strength was expressed as ratios ER/IR and eccER/IR both sexes showed a lower ratio for eccER/IR in national players (0.95 ± 0.22 and 0.95 ± 0.23) compared to regional players (1.01 ± 0.32 and 1.07 ± 0.29) for male and female players, respectively. In conclusion, this paper presents a tennis-specific normative database for shoulder rotation strength and ROM in adolescent male and female competitive players. The key points in this evaluation are strength values normalized to body mass, intermuscular ratios, and TROM.

## Introduction

Tennis is an intermittent sport in which players need to master a range of demands of physical components, such as aerobic and anaerobic capacity, linear sprint and change-of-direction speed, agility, and muscle power to reach the highest levels of performance (Fernandez-Fernandez et al., 2018; Björklund et al., 2020). Furthermore, male and female professional tennis players perform the serve motion to a larger extent compared with their younger counterparts (i.e., high performance junior players) (Myers et al., 2016). At high-performance junior levels, players perform per match an average of 60–70 serves at a speed range from 145 to 160 kph (Kovalchik and Reid, 2017). From a biomechanical point of view the shoulder moves into an external rotation (ER) of 170 degrees in the late cocking phase, followed by a shoulder internal rotation (IR) taking place after ball impact at 2,420 and 1,370°/s for male and female players, respectively (Fleisig et al., 2003). In addition, a strong positive correlation between peak serve speed and shoulder IR and ER strength has been shown (Hayes et al., 2021), highlighting the link between good shoulder capacity and sport-specific performance. The competitive adolescent tennis player has previously been reported to have age related increase in shoulder strength, decreased rotational range of motion (ROM) in IR, increased ER ROM, and decreased total ROM (TROM) in the dominant arm (DA) used for the overhead serve motion, parallel to the growth process (Cools et al., 2014b; Gillet et al., 2017; Fernandez-Fernandez et al., 2019).

Investigations of adolescent tennis players competing on the highest level have reported injury rates of 1.2–2.8 injuries per 1,000 h played (Pluim et al., 2016; Gescheit et al., 2019; Moreno-Pérez et al., 2019) and amongst these injury rates, overuse injuries have been reported to be the most common health complaint among junior tennis players with a weekly prevalence of 12.1%, compared to acute injuries with a weekly prevalence of 3% (Pluim et al., 2016). Moreover, a considerable proportion of these overuse injuries are in the dominant shoulder, with an incidence of 8.2 injuries per 1,000 playing hours in tennis matches, accounting for 15.9% of all overuse injuries in high-performance junior tennis players (Pluim et al., 2006; Fu et al., 2018).

Factors of importance for the origin of injury in overhead athletes such as handball and tennis players, and across all age groups have been reported to be, imbalances in terms of low intermuscular ratios between ER/IR strength, ER weakness, decreased IR ROM and decreased TROM of the shoulder (Saccol et al., 2010; Andersson et al., 2017; Achenbach and Luig, 2020; Asker et al., 2020).

Since adolescent athletes are not yet fully developed, and early onset of adaptations occur, continuous assessments at shoulder level throughout puberty are crucial for the guidance and optimization of the tennis players training regime (Oliver et al., 2020). In view of clinical assessment, field-friendly measurement tools which are reliable, valid, and cost-effective like the hand-held dynamometer (HHD) and smartphone inclinometer have been proposed (Cools et al., 2014b; Mejia-Hernandez et al., 2018). Furthermore, a recent meta-analysis showed good absolute reliability for HHDs in shoulder internal and external rotator strength assessment reinforcing previous studies (Chamorro et al., 2021). Previously, a general reference database based on HHD measurements for tennis players has been published, however, the study sample was relatively small (*n* = 65), the players were 18–50 years old, and a recreational playing level constituted the subjects represented (Cools et al., 2016).

Therefore, the primary aim with our study is to provide normative values at shoulder level for isometric and eccentricER (eccER) strength, intermuscular ratios ER/IR, and rotational ROM for the adolescent competitive tennis player. In addition, we hypothesized that sex, age, side, and level of play differences would exist between test values.

## Materials and Methods

### Participants

Three hundred and one adolescent competitive tennis players, 176 males and 125 females, mean age 14.6 (±2.0) and 14.4 (±2.0) years, respectively, volunteered to participate in the study. Recruitment of the players was performed via all seven tennis regions in Sweden and included both national (*n* = 50) and regional (*n* = 251) level players. A baseline questionnaire was filled out before the testing and an informed consent form was read and signed by the players, if under 15 years of age, the players' legal guardian read and signed the consent form. Inclusion criteria were (1) competitive level of at least regional level in Sweden (2) minimum of 8 h of total training volume per week on average. Subjects were excluded if they had shoulder surgery or dislocation the last 6 months. Subclassification of the tennis players was made based on sex, age, and level of play. Classification for age was divided into (a) 14 years and under and (b) 15 years and over, in accordance with the competing system of Tennis Europe and the International Tennis Federation^1^,^2^ In addition, level of play was divided into regional and national level based on the high-performance program conducted by the Swedish Tennis Association^3^.

### Ethical Statement

The study was in accordance with the declaration of Helsinki and preapproved by the Regional Ethical Review Board, Stockholm, Sweden (approval no. 2012/1731/2).

### Testing Procedure

Prior to the testing procedure, the players body mass was assessed on a digital scale, shoes and heavy clothing were removed and the result was recorded to the nearest decimal fraction. A supervised and standardized warm-up program for 10 min was performed, consisting of several multiplanar shoulder movements including three light elastic band exercises performed 2 sets x 15 repetitions/side and two general flexibility exercises for the thoracic spine and the upper limb was performed 2 sets x 10 repetitions/side by the players prior to the testing. The assessments consisting of both strength and mobility in a standardized field-based setting were performed on a single visit by three teams of three assessors per team, all teams were trained prior to the testing by an experienced clinician and user of the HHD.

### Glenohumeral Muscle Strength

For all strength measurements, the MicroFET© HHD was used (MicroFet 2, Hoggan Health Industries Inc., Biometrics, The Netherlands). To control the testing procedure for learning effects and fatigue, the order of the tests was randomized between sides. The protocol consisting of six different strength tests were performed in both the DA and non-dominant arm (NDA) independently: (1 and 2) isometric shoulder strength of IR and ER at shoulder level with 0° of abduction (ABD), (3 and 4) isometric shoulder strength of IR and ER in 90° ABD, (5) isometric shoulder strength of ABD in the scapular plane, (6) eccER shoulder strength testing in an abducted position from 90° of ER to 0° of ER were included (Clarsen et al., 2014; Cools et al., 2014a). Strength measurements were recorded in Newton (N) by the HHD, and the second examiner registered the test value into the test-protocol. Each test was repeated two times with a pause in between trials of 20 s (Cools et al., 2014a).

**Figure 1:**
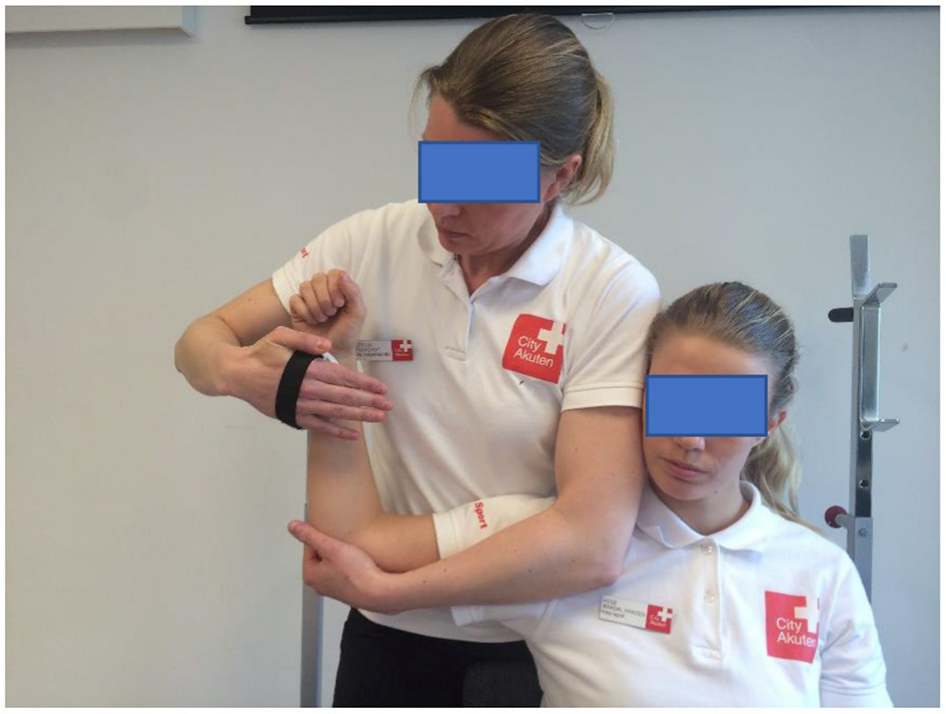
Measurement of isometric muscle strength of the internal rotators using a hand-held dynamometer (CompuFET, Hoggan Health Industries Inc., Groningen, The Netherlands).

**Figure 2:**
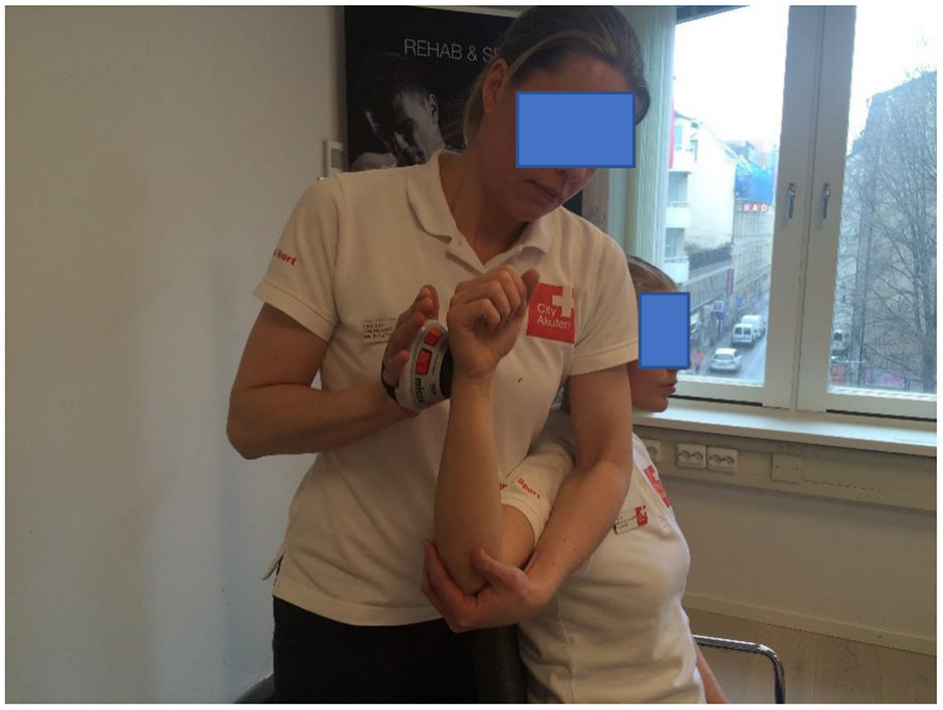
Measurement of isometric muscle strength of the external rotators and starting position for eccentric strength measurement using a hand-held dynamometer (CompuFET, Hoggan Health Industries Inc., Groningen, The Netherlands).

Isometric IR and ER testing took place in a seated position, the arm was supported in shoulder position at 90° of ABD and neutral rotation (illustrative Figures 1, 2) (Cools et al., 2014a). Isometric ABD was measured with the player in a standing position with the arm held 30° of ABD in the scapular plane (Clarsen et al., 2014). For all strength tests the participants were asked to build up their force gradually to a maximum voluntary effort over a 2-s period and hold the maximal voluntary effort for 5 s, two sets of trials were performed, and the average value was used for calculation.

The eccER shoulder strength testing was performed in a seated position, starting in 90° of ABD and 90° of ER, with the arm supported by the examiner, the HHD was positioned 2 cm proximal of the processus styloideus ulnae and placed on the dorsal side of the forearm (Johansson et al., 2015b). The participant performed a resisted ER, and the examiner moved the arm into IR for 90° in 3 s (illustrative Figure 2).

All testing procedures described above demonstrated good to excellent intra- and inter-rater reliability (Cools et al., 2014a; Johansson et al., 2015b).

### Glenohumeral Internal- and External Rotation Range of Motion

**Figure 3:**
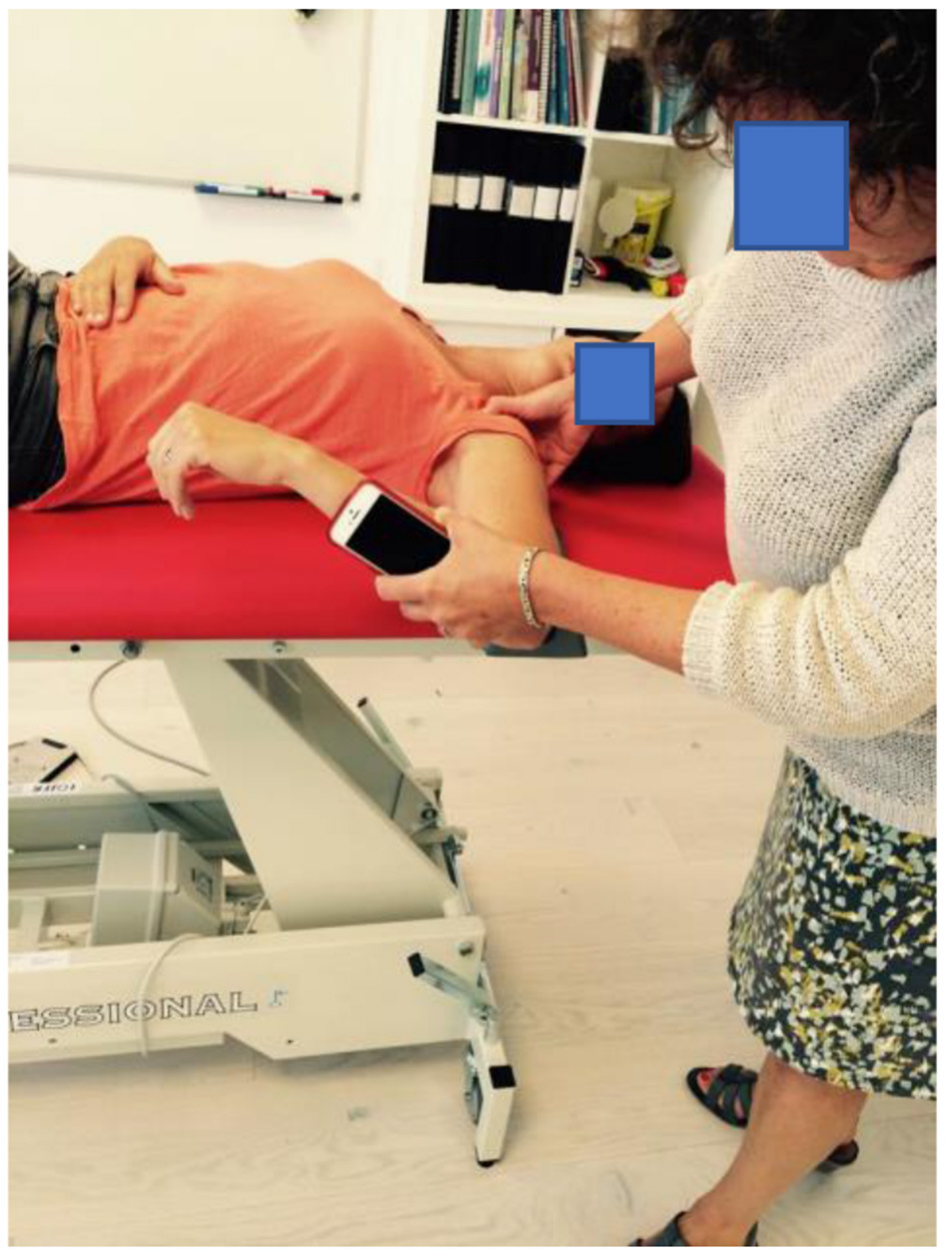
Measurement of glenohumeral internal-rotation range of motion using a smartphone inclinometer application (GetMyROM (version 1.0.3; Interactive Medical Productions, Hampton, NH, USA).

Shoulder IR and ER passive ROMs were measured for the dominant and non-dominant shoulder using a smartphone inclinometer app, GetMyROM (version 1.0.3; Interactive Medical Productions, Hampton, NH, USA) and following the methods previously described in the literature (Mejia-Hernandez et al., 2018). Participants were supine with their shoulders positioned in 90° of ABD in the coronal plane. Measurements for IR and ER ROM were performed in the plane of ABD, and a small towel roll was used to maintain the position of the humerus. The inclinometer was positioned on the player's forearm (illustrative Figure 3). Two examiners performed the test, examiner one took the shoulder to full ROM without using overpressure, scapular movement was controlled by palpating the coracoid process, examiner two read and noted the ROM in IR and ER. These procedures have previously shown good test-retest reliability (Cools et al., 2014b), and excellent intra- and interrater reliability (Cools et al., 2014b).

### Statistical Analysis

To analyze the difference in isometric and eccentric strength and the intermuscular ratios a general linear model two-way analysis of variance (ANOVA) for repeated measures, in which the within-subject factor was side (two levels), and the between-subject factors were sex (two levels), level of play (three levels), and age category (three levels). The eccentric and isometric strength measurements were analyzed both in Newton and as normalized by body mass. The following isometric muscle strength ratios were also calculated: ER/IR at 0–0° and 90–90° and the functional ratio eccER/isometric IR. For mean calculations and ratios between different measurements, the individual measurements were treated separately and no mean values of the two measurements per individual were used.

To assess the reliability of the strength measurements, the Intraclass correlation coefficients (ICC) (3.1), two-way mixed model, agreement) were calculated, along with their 95% confidence intervals, over the two measurements taken. Standard error of measurement (SEM) was calculated as SD^*^√ (1- ICC), where SD is the standard deviation of the measurement. Minimum detectable change (MDC95) was calculated as SEM^*^1.96^*^√2.

Interaction effects, as well as main effects were explored. In case of absence of any significant interactions, main effects for (age, side, sex, and level of play) were analyzed. In the ANOVA a *p* < 0.05 was considered statistically significant. *Post-hoc* analyses were performed using a Bonferroni test when a significant difference was found with ANOVA. For the ANOVA analysis, the mean value was taken of the two measurements done for each subject. In case of main effects for sex and side, no *post-hoc* tests were necessary.

Analysis was done in R (version 3.6.1, R Core Team, 2019). R: A language and environment for statistical computing. R Foundation for Statistical Computing, Vienna, Austria) and Stata, version 15 (StataCorp. 2017. Stata Statistical Software: Release 15. College Station, TX: StataCorp LLC.).

## Results

Anthropometric data, training background and level of play are summarized in Table 1.

**Table 1:** Characteristic data of the tennis players in the study (*n* = 301).

	**Female** **(*n* = 125)**	**Male** **(*n* = 176)**
Age year, mean (SD)	14.4 (2.0)	14.6 (2.0)
Height cm, mean (SD)	165.2 (7.6)	173.0 (12.3)
Weight kg, mean (SD)	55.1 (9.6)	60.6 (14.1)
Number of players–Age range 14 and under (%)	74 (59)	94 (53)
Number of players–Age range 15 and over (%)	51 (41)	82 (47)
Level of Play–National squad (%)	28 (22)	22 (12.5)
Level of Play–Regional squad (%)	97 (78)	154 (87.5)
Total training h/week, National squad, mean (SD)	14.8 (5.3)	16.1 (3.7)
Total training h/week, Regional squad, mean (SD)	11.3 (5.5)	12.5 (4.2)

*SD, standard deviation*.

*Total Training is the Sum of Tennis and Fitness but no other sports*.

The results of the reliability study measurement are summarized in Table 2.

**Table 2:** Intraexaminer reliability (ICC_3,k_) with their 95% CI, SEM and MDC (N) for the range of motion, isometric, and eccentric strength testing of the rotator cuff.

**Position**	**Dominant arm**	**SEM**	**MDC (*N*)**	**Non-dominant arm**	**SEM**	**MDC (*N*)**
Passive max ROM IR	0.90 (0.87–0.92)	3.87	10.73	0.91 (0.89–0.93)	3.85	10.68
Passive max ROM ER	0.91 (0.89–0.93)	3.81	10.55	0.87 (0.84–0.90)	4.51	12.50
Isom IR 0–0	0.95 (0.94–0.96)	9.01	24.97	0.96 (0.95–0.97)	7.31	20.27
Isom ER 0–0	0.94 (0.93–0.95)	5.54	15.36	0.93 (0.91–0.95)	5.98	16.58
Isom IR 90–90	0.95 (0.93–0.96)	9.37	25.98	0.93 (0.90–0.95)	9.29	25.76
Isom ER 90-90	0.94 (0.92–0.95)	5.64	15.62	0.92 (0.90–0.94)	6.52	18.06
Ecc ER 90–90 to 90–0	0.94 (0.93–0.95)	7.58	21.01	0.92 (0.90–0.93)	8.95	24.80
Isom ABD 30°	0.95 (0.94–0.96)	6.78	18.80	0.94 (0.92–0.95)	7.29	20.22

*ROM, range of motion; Max, maximal; ER, external rotation; IR, internal rotation; Ecc, eccentric; Isom, isometric; 0–0°, 0° abduction 0° external rotation, 90–90° 90° abduction 90° of external rotation (ABER); ABD, abduction; SEM, standard error of measurement; MDC, minimal detectable change; N, Newton*.

Supplementary Tables 1, 2 show the results of the isometric strength, eccentric strength, and ER/IR strength ratios and eccER/IR strength ratios testing for male and female players, respectively, and (a) across all ages, (b) divided by age, and (c) divided by level of play.

Tables 3, 4 display the rotational ROM results of male and female players, respectively, and (a) across all ages, (b) divided by age, and (c) divided by level of play. Summarized results for the ANOVA repeated measures statistical analysis and *post-hoc* tests, are displayed in Table 5. Furthermore, in Appendix 1, ROM, strength values and intermuscular ratios are presented for male and female players as percentiles and in Appendix 2, a comparison between male and female players shoulder strength are presented (Figures 4, 5 in Appendix 2).

**Table 3:** Descriptive analysis (means and SDs) of the results of the Range of Motion (ROM) for the male (*n* = 176) subjects.

	**ROM IR**		**ROM ER**		**Total ROM**	
	**D**	**ND**	**D**	**ND**	**D**	**ND**
**MALE**						
**(a) Across all ages**						
Mean	57.2	65.4	98.4	91.3	155.6	156.7
SD	12.2	11.6	12.5	12.8	17.4	17.7
**AGE**						
**(b) Divided per age category**						
Under 14 years (*n* = 94)						
Mean	58.8	66.3	97.3	91.5	156.2	157.8
SD	11.8	10.2	12.5	12.5	16.1	16.7
Over 15 years (*n* = 82)						
Mean	55.4	64.5	99.6	91.1	155.0	155.5
SD	12.5	13.0	12.4	13.1	18.7	18.8
**LEVEL OF PLAY**						
**(c) Divided per level of play**						
National players (*n* = 22)						
Mean	56.8	63.2	102.5	97.0	159.3	160.2
SD	10.7	9.4	14.2	10.1	15.0	13.2
Regional Players (*n* = 154)						
Mean	57.3	65.7	97.8	90.5	155.1	156.2
SD	12.4	11.9	12.1	13.0	17.6	18.3

*ER, external rotation; IR, internal rotation; ROM, range of motion; D, dominant; ND, non-dominant*.

**Table 4:** Descriptive analysis (means and SDs) of the results of the Range of Motion (ROM) for the female subjects (*n* = 125).

	**ROM IR**		**ROM ER**		**Total ROM**	
	**D**	**ND**	**D**	**ND**	**D**	**ND**
**FEMALE**						
**(a) Across all ages**						
Mean	61.5	71.5	98.5	93.5	160.0	165.0
SD	11.6	13.4	13.1	12.5	17.0	17.2
**AEG**						
**(b) Divided per age category**						
Under 14 years (*n* = 74)						
Mean	61.2	71.5	98.2	93.7	159.4	165.2
SD	11.1	13.9	12.2	12.3	16.4	17.6
Over 15 years (*n* = 51)						
Mean	62.0	71.5	98.8	93.3	160.9	164.8
SD	12.2	12.7	14.2	12.9	17.7	16.7
**LEVEL OF PLAY**						
**(c) Divided per level of play**						
National players (*n* = 28)						
Mean	60.3	70.7	99.4	97.3	159.7	168.0
SD	13.2	13.8	9.5	10.6	17.9	14.8
Regional Players (*n* = 97)						
Mean	61.9	71.7	98.2	92.4	160.1	164.2
SD	11.0	13.3	13.9	12.9	16.7	17.7

*ER, external rotation; IR, internal rotation; ROM, range of motion; D, dominant; ND, non-dominant*.

**Table 5:** Results from the repeated measures ANOVA for all variables.

		**Isom**		**Isom**		**Isom**		**Isom ER**		**Ecc ER**		**Isom ABD**		**Isom ER/**		**Isom ER/**		**EccER/isom**		**ROM IR**	**ROM ER**	**ROM** **total**
		**IR 0–0**		**ER 0–0**		**IR 90–90**		**90–90**						**IR 0–0**		**IR 90–90**		**IR 90–90**				
		**ABS**	**NORM**	**ABS**	**NORM**	**ABS**	**NORM**	**ABS**	**NORM**	**ABS**	**NORM**	**ABS**	**NORM**	**ABS**	**NORM**	**ABS**	**NORM**	**ABS**	**NORM**	**ABS**	**ABS**	**ABS**
	**Three-way-interaction**																					
Three-way-interaction	Gender x Age x Side	NS	NS	NS	NS	NS	NS	NS	NS	NS	NS	NS	NS	NS	NS	NS	NS	NS	NS	NS	NS	NS
	Side x Age x Level of play	^*^	NS	^*^	NS	NS	NS	NS	NS	NS	NS	NS	NS	NS	NS	NS	NS	NS	NS	NS	NS	NS
	Gender x Age x Level of play	NS	NS	NS	NS	NS	NS	NS	NS	NS	NS	NS	NS	NS	NS	NS	NS	NS	NS	NS	NS	NS
	Gender x Side x Level of play	NS	NS	NS	NS	NS	NS	NS	NS	NS	NS	NS	NS	NS	NS	NS	NS	NS	NS	NS	^*^	NS
	**Two-way-interaction**																					
Two-way-interaction	Gender x Age	^***^	NS	^***^	^*^	^***^	NS	^***^	NS	^***^	NS	^***^	^***^	NS	^*^	NS	NS	NS	^*^	NS	NS	NS
	Gender x Side	NS	NS	NS	NS	NS	NS	NS	NS	NS	NS	NS	NS	NS	NS	NS	NS	NS	NS	NS	^**^	^**^
	Gender x Level of play	NS	NS	NS	NS	NS	NS	NS	NS	NS	NS	NS	NS	NS	NS	NS	NS	NS	NS	NS	NS	NS
	Age x Side	NS	NS	NS	NS	NS	NS	NS	NS	NS	NS	NS	NS	NS	NS	NS	NS	NS	NS	NS	NS	NS
	Age x Level of play	^**^	^*^	NS	NS	^**^	NS	^*^	NS	NS	NS	^*^	NS	^*^	^*^	NS	NS	NS	^*^	NS	NS	NS
	Side x Level of play	NS	NS	NS	NS	NS	NS	NS	NS	NS	NS	NS	NS	NS	NS	NS	NS	NS	NS	^**^	NS	^*^
	**Main effects**																					
Main effects	Age	^***^	NS	^***^	NS	^***^	^*^	^***^	NS	^***^	^*^	^***^	^*^	NS	^***^	NS	^***^	NS	^***^	NS	NS	NS
	Side	NS	NS	NS	NS	^*^	NS	NS	NS	^*^	NS	NS	NS	NS	NS	NS	NS	NS	NS	^***^	NS	NS
	Gender	^***^	^***^	^***^	^**^	^***^	^***^	^***^	^**^	^***^	^***^	^***^	^***^	^**^	^***^	NS	^*^	NS	^*^	^**^	NS	^*^
	Level of play	NS	NS	NS	NS	NS	NS	NS	NS	NS	NS	NS	NS	NS	NS	NS	NS	NS	^*^	NS	NS	NS
*Post-hoc* test: Bonferroni																						
Two-way-interaction	Gender x Age	^***^	NS	^***^	NS	^***^	NS	^***^	NS	^***^	NS	^***^	^**^	NS	NS	NS	NS	NS	^*^	NS	NS	NS
	Gender x Side	NS	NS	NS	NS	NS	NS	NS	NS	NS	NS	NS	NS	NS	NS	NS	NS	NS	NS	NS	^*^	^*^
	Gender x Level of play	NS	NS	NS	NS	NS	NS	NS	NS	NS	NS	NS	NS	NS	NS	NS	NS	NS	NS	NS	NS	NS
	Age x Side	NS	NS	NS	NS	NS	NS	NS	NS	NS	NS	NS	NS	NS	NS	NS	NS	NS	NS	NS	NS	NS
	Age x Level of play	^**^	^*^	NS	NS	^*^	NS	NS	NS	NS	NS	NS	NS	NS	NS	NS	NS	NS	NS	NS	NS	NS
	Side x Level of play	NS	NS	NS	NS	NS	NS	NS	NS	NS	NS	NS	NS	NS	NS	NS	NS	NS	NS	^*^	NS	NS

* *Significant at 0.05 level*.

** *Significant at 0.1 level*.

*** *Significant at 0.01 level*.

*NS, not significant; ABS, absolute strength values; NORM, normative values to body weight*.

Present results show significant differences for age and sex in all six strength tests. However, the three calculated intermuscular strength ratios based on the strength testing showed a lower ratio in the male players compared to the female players, most likely due to the strong internal rotators seen in male players. Overall, the reported test values of strength in the DA were higher than in the NDA, however, the NDA showed higher values of intermuscular ratios. Moreover, national level players are stronger than their regional counterparts with male players showing the largest difference. Male players increase their strength values with age but when normalized to body mass the increase remains only in isometric ABD strength. In addition, male players are stronger than female players even when strength values are normalized to body mass. Female players increase their strength with age in absolute values but when normalized to body mass the strength results show a decrease. The eccER/isometric IR ratio revealed a decrease with increased age and national players reporting a lower ratio than regional players.

A two-way interaction effect was significant for age x sex and age x playing level for the six strength tests plus the intermuscular strength ratio: isometric ER/IR 0–0 and the eccER/isometric IR 90–90, reflecting that there are differences for age but not for both sexes/playing level or there are differences for sexes/playing level but not for different age groups.

Analysis for three-way interaction effects showed significant strength differences for side x age x playing level in isometric IR and ER at neutral position 0°- 0°. A 3-way interaction effect was also significant for sex x side x playing level regarding ER ROM. Neither of the 3-way interaction effects showed a significant difference in the *post-hoc* Bonferroni analysis. However, the two-way interaction effect, sex x age, remained significant in all strength tests in the *post-hoc* Bonferroni analysis. Lastly, the same significant outcome was noted for isometric IR strength at 0–0 and 90–90 position.

The ER ROM showed significant sex x side x playing level in the three-way interaction effect with male national players showing increased ER ROM in the DA compared to male regional players as well as compared to all female players. Furthermore, the TROM and ER ROM displayed significant differences in the two-way interaction effect for sex x side, with the DA showing increased ER and decreased TROM for both male and female players. Finally, the side x playing level in the two-way interaction effect revealed a significant difference in the IR and TROM in both sexes. The *post-hoc* Bonferroni analysis confirmed the previous results in ROM presented in the two-way interaction effects for side x playing level regarding IR, and sex x side regarding TROM and ER.

## Discussion

The main purpose of the study was to establish normative values at shoulder level for isometric and eccentric strength, as well as rotational ROM for the adolescent competitive tennis player. The main results showed, age and sex differences in the isometric as well as the eccER shoulder strength in adolescent competitive tennis players. Secondly, the DA is stronger than the NDA and national players are stronger than their regional counterparts. Thirdly, age, sex, and playing level differences for the intermuscular strength ratio: isometric ER/IR 0–0 and the eccER/isometric IR 90–90 were seen. Finally, both male and female players showed a difference in IR ROM of the DA compared to the NDA, however, male national players revealed higher values in ER ROM in the DA compared to regional players.

### Shoulder Rotational Strength

Present results showed that glenohumeral strength values were higher in male players compared with female players, which is in line with previous studies of young tennis players (Cools et al., 2014b; Gillet et al., 2017; Fernandez-Fernandez et al., 2019). Both male and female players are stronger in the dominant side when compared to the non-dominant side confirming the results of a previous study on adolescent tennis players (Cools et al., 2014b) and highlighting the asymmetric nature of the sport (Rogowski et al., 2008). The asymmetry may be a result of playing tennis rather than handedness (Rogowski et al., 2008). Several asymmetric findings in the dominant shoulder have been reported in literature, including both male and female players, adolescent, and professional players, reporting clinical infraspinatus atrophy, early signs of tendinosis in the infraspinatus and supraspinatus tendons, ROM deficits and increased strength in the dominant shoulder (Johansson et al., 2015a; Young et al., 2015; Gillet et al., 2017; Ellenbecker et al., 2020). Therefore, it seems inevitable to not be affected in the dominant shoulder by these high numbers of repetitions. However, if these adaptations are to be considered as normal adaptations, risk factors or to be entitled as maladaptation's needs further investigation. Regarding the development of strength levels, results for both sexes increase with age as seen in other studies (Cools et al., 2014b; Gillet et al., 2017; Fernandez-Fernandez et al., 2019). However, when normalized to body mass, our study shows that the strength values of the female players are leveling out comparing the age group 14 years and under with the age group 15 years plus, whilst male players still increase their relative strength throughout adolescence. In view of rapid anthropometry changes during puberty and the onset of hormones girls are more likely to have a tougher challenge to sustain their strength normalized to body mass in comparison with the boys. From the performance and prevention perspective, it would be recommended that tennis players on the international level in transition from Tennis Europe under 14 to junior ITF 15–18 years with an increased level of competition, improve their shoulder strength within this timeframe. Furthermore, a long-term athlete development strategy is recommended also in the perspective of strength development during this period provided that sufficient time are given to the players by the coaches in the training plan to improve strength.

### Playing Level and Strength

In view of the playing level perspective, national players presented higher strength values overall compared to their regional level peers. The difference was especially observed in the isometric IR 90–90 position reporting higher values (134.8 ± 33.4 vs. 126.0 ± 46.0 N and 107.3 ± 21.6 vs. 96.5 ± 25.7 N) in the DA, for male and female players, respectively. Considering that IR at high shoulder elevation angles takes place during the tennis serve motion, the difference in strength results in the IR 90–90 position may reflect the higher volume of sport-specific training seen in national players compared to regional players. Consequently, this repetitive motion in shoulder IR is most likely to develop IR strength over time as the service motion to a great extent engage the internal rotators of the shoulder complex (Escamilla and Andrews, 2009).

### Eccentric External Rotation Strength

This is the first study to investigate eccER shoulder strength in a large cohort of adolescent competitive tennis players using an HHD. In our study male players showed eccER strength increase with age, higher values in the DA and a difference between national and regional players. However, female players showed only an improvement in eccER strength with age, no difference was found either for side or level of play. The eccER strength normalized to body mass for male and female players were in our study 2.0 and 1.8 N/kg, respectively. In comparison with a similar cohort in age, investigating competitive adolescent handball players, results were similar, reporting 1.9 and 1.7 N/kg for male and female players, respectively (Asker et al., 2020). Furthermore, a sample of 65 recreational tennis players in the age group 18–25 years reported normalized eccER strength values of 2.2 and 2.1 for male and female subjects, respectively (Cools et al., 2016). In summary, the low ratios in adolescent athletes may reflect the challenge to develop eccER strength in overhead athletes during puberty no matter the sport. In addition, recreational players may not be as fatigued in the shoulder as competitive players regardless of age and therefore reporting higher values in eccER.

### Intermuscular Ratios in Dominant and Non-dominant Arm

Results revealed in all three calculations for both male and female players a lower ratio in the DA compared to the NDA. Our results showed an eccER/IR isometric ratio 90–90° of 1.00/1.13 for male players and 1.05/1.23 for female players in the DA and NDA, respectively. These results are in line with previously reported ratios, although reported in an older sample (27.6 ± 8.4 years), results being lower in the DA of tennis player (Cools et al., 2016). In addition, similar results have been reported in a younger cohort of tennis players, reporting lower isometric ER/IR intermuscular ratios in the DA (Gillet et al., 2017; Fernandez-Fernandez et al., 2019). This lower ratio may be explained by several factors. Firstly, the DA is subject to a high number of repetitions and therefore the shoulder internal and external rotator muscles are most likely to be fatigued. Secondly, when calculating ratios, we need to consider the IR strength being more developed in the DA and especially in male players, due to the high numbers of serving compared to the NDA not being used in the overhead motion, therefore affecting the ER/IR ratio calculation. Moreover, intermuscular ratios being lower in the DA of adolescent athletes is a phenomenon seen in other sports as well, such as handball and baseball (Trakis et al., 2008; Asker et al., 2020).

### Level of Play and Intermuscular Ratios

In view of level of play, male and female national players in our study presented a lower ratio for the eccER/IR strength ratio compared to regional players. In the light of national players being stronger especially in isometric IR 90–90° this might be the explaining factor for the lower ratio, on the other hand, it should also be highlighted that with more hours per week in match, practice, and fitness, a potential fatigue of the shoulder internal and external rotator muscles may occur. In view of ER/IR ratios, at both positions (0 and 90°) the male and female players regardless of level in our study performed a ratio <0.75. Therefore, it may be of special importance to improve the ER strength of the shoulder since decreased ER strength has been previously reported to be associated with injury in the adolescent and elite handball player (Saccol et al., 2010; Asker et al., 2020). In addition, low ratios of ER/IR have also been reported to increase injury risk in the professional baseball pitcher (Byram et al., 2010).

### Range of Motion

In overall the ROM results revealed a difference between sexes with male players showing less IR and TROM compared to female players, both sexes showing less IR and increased ER in the DA compared to the NDA. In addition, female players displayed a decrease in TROM in the DA compared to the NDA. Male players showed a decrease in IR ROM and increase in ER ROM with age. However, TROM was not affected, therefore the results suggesting a shift in the rotational range to be the explanation (Whiteley et al., 2009). In the perspective of level of play, male national players displayed higher ER ROM and higher TROM compared to regional players, IR remained the same for both playing levels. Female players showed no difference either in age or in level of play. This increase in ER ROM in male national players may be a consequence of more training and match volume and thereby a greater number of overhead motions enhancing ROM in ER (Myers et al., 2016). In addition, based on practitioner experience it might reflect a traditional training paradigm with male players practicing more serves on the national level compared with female national players practicing more from the baseline. Previous studies of ROM in tennis players have reported a decrease in IR with an increase in age, TROM remains the same with age, however, a shift is apparent with an increase in ER in combination with a decrease in IR (Cools et al., 2014b; Gillet et al., 2017; Fernandez-Fernandez et al., 2019; Moreno-Pérez et al., 2019).

### Strength and Limitations

The strengths of the study were firstly the large cohort including 301 adolescent competitive national and regional tennis players, therefore representing most of the available players. Secondly, the cohort included both male and female players enabling the possibility to investigate and compare the two sexes. Thirdly, the reliability of the clinical measures performed with a HHD was good to excellent, with ICC ranging between 0.87–0.91, SEM 3.81–4.51, and MDC 10.55–12.50, consequently, making the results clinically relevant (Cools et al., 2014a; Johansson et al., 2015b). In addition, the smartphone app used for ROM assessments has also proven to be reliable in the clinical setting (Mejia-Hernandez et al., 2018). Lastly, the present study and its results have high levels of ecological validity and may offer a starting point to suggest practical applications to tennis-specific fitness and prevention training.

However, this study has some limitations. Firstly, it should be highlighted that the reference values suggested here only is comparable in the clinical setting provided that the clinician is using the HHD as an assessment tool. Moreover, no standardized threshold in force output was used between the two trials, potentially this could affect the results if one of the trials being a submaximal effort or if there was a learning effect. This would be recommended for future assessments, however, in view of the large cohort it was not possible due to time management. In addition, when assessing stronger players, it may require stronger assessors to resist the strength of the player's push and obtain reliable results (Croteau et al., 2021). Although assessing shoulder strength using an HHD in a field-based setting is reliable, the results should be treated with caution due to high threshold for reliable measures, especially in adolescents (Møller et al., 2018). Furthermore, no external fixation was used during the assessments due to practical and clinical relevance, this might have influenced our results. However, external fixation in the field is not very practical due to the extra time needed to set-up the assessment procedure, therefore, our protocol was developed to be more clinically relevant. The end range in the ROM measurements was determined by subjective criteria, based on clinical skills, but was not objectively controlled. Therefore, the results may be affected by the skills of the examiner. Finally, although this is a large cohort (*n* = 301), future studies should be focused on international multicenter studies due to the differences in training volume between countries. However, all things considered this normative database may help all stakeholders involved in the adolescent tennis player to make better decisions regarding rehabilitation, return to play and high-performance.

## Conclusion

This is the first paper to present specific isometric and eccER shoulder strength values measured with a HHD and shoulder rotational ROM data based on a large (*n* = 301) cohort of adolescent male and female competitive tennis players. Our most important findings of the study were age and sex differences in the isometric as well as in the eccER shoulder strength. In view of performance the study highlights the gain in shoulder ER ROM and the need for developing strength throughout puberty especially in female players. Finally, the potential risk factor reported as ER weakness is evident also in our cohort.

## Data Availability Statement

The raw data supporting the conclusions of this article will be made available by the authors, without undue reservation.

## Ethics Statement

The study was in accordance with the declaration of Helsinki and preapproved by the Regional Ethical Review Board, Stockholm, Sweden (approval no. 2012/1731/2). Written informed consent to participate in this study was provided by the participants' legal guardian/next of kin.

## Author Contributions

FJ, MA, JF-F, and AC designed the study. FJ, AM, and MA were part of the data collection. FJ, AW, and AC analyzed the data and prepared the manuscript. All authors read and approved the final manuscript, contributed to the article, and approved the submitted version.

## Funding

This study was funded by the Swedish Naprapathic Association.

## Conflict of Interest

The authors declare that the research was conducted in the absence of any commercial or financial relationships that could be construed as a potential conflict of interest.

## Publisher's Note

All claims expressed in this article are solely those of the authors and do not necessarily represent those of their affiliated organizations, or those of the publisher, the editors and the reviewers. Any product that may be evaluated in this article, or claim that may be made by its manufacturer, is not guaranteed or endorsed by the publisher.

## Footnotes

^1^ https://www.itftennis.com/en/itf-tours/world-tennis-tour-juniors/

^2^ https://www.tenniseurope.org/page/12174/Official-Events

^3^ https://www.tennis.se/elit-och-landslag/juniorlandslag/
