# Supplementary material for: Eccentric and Isometric Shoulder Rotation Strength and Range of Motion: Normative Values for Adolescent Competitive Tennis Players
Source: Front Sports Act Living. 2022 Feb 17;4:798255. doi: 10.3389/fspor.2022.798255 (PMC8891455; doi:10.3389/fspor.2022.798255)

**APPENDIX 2.**

FIGURE 4. Shoulder strength in the dominant arm for male (n=176) and female players (n=125).
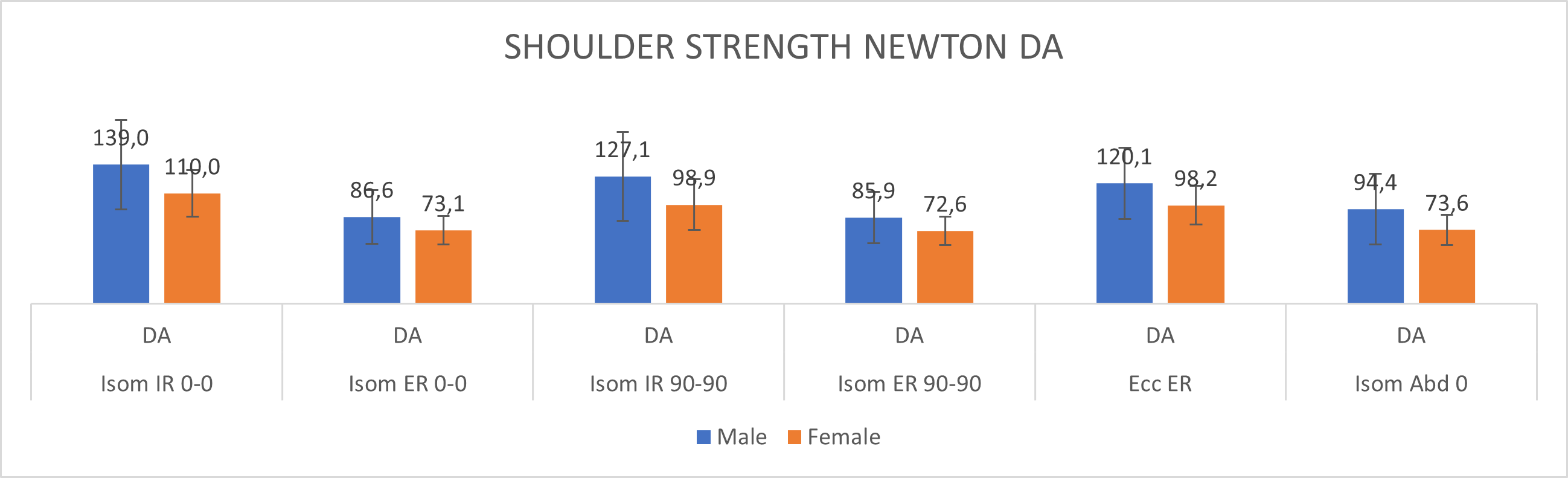


FIGURE 5. Shoulder strength in the non-dominant arm for male (n=176) and female players (n=125).


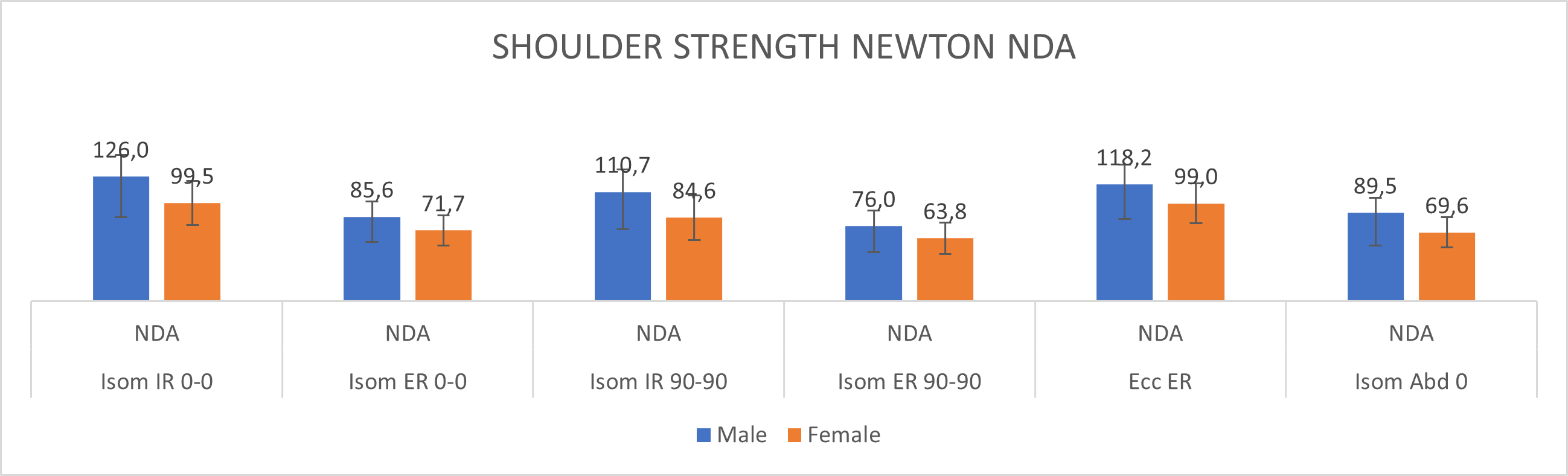

Supplement: Supplementary file 2 [file Table_2.docx]
